# Increased Risk of Pneumonia in Patients Receiving Gonadotropin-Releasing Hormone Agonists for Prostate Cancer

**DOI:** 10.1371/journal.pone.0101254

**Published:** 2014-06-27

**Authors:** Shiu-Dong Chung, Shih-Ping Liu, Herng-Ching Lin, Li-Hsuan Wang

**Affiliations:** 1 Division of Urology, Department of Surgery, Far Eastern Memorial Hospital, Banciao, New Taipei City, Taiwan; 2 School of Medicine, Fu-Jen Catholic University, New Taipei City, Taiwan; 3 Sleep Research Center, Taipei Medical University Hospital, Taipei, Taiwan; 4 Department of Urology, National Taiwan University Hospital and College of Medicine, Taipei, Taiwan; 5 School of Health Care Administration, Taipei Medical University, Taipei, Taiwan; 6 School of Pharmacy, Taipei Medical University, Taipei, Taiwan; 7 Department of Pharmacy, Taipei Medical University Hospital, Taipei, Taiwan; National Health Research Institutes, Taiwan

## Abstract

**Background:**

This study aimed to investigate the relationship between the use of gonadotropin-releasing hormone (GnRH) agonists and subsequent risk of pneumonia in patients with prostate cancer (PC) using a population-based dataset.

**Methods:**

We obtained the data from Taiwan's Longitudinal Health Insurance Database 2000. We included 2064 PC in this study. Of the sampled PC patients, 1207 received treatment with GnRH agonists. We individually traced each PC patient for a 1-year period to identify those who were hospitalized with pneumonia. We performed a Cox proportional hazard regression to explore the association between the use of GnRH agonists and the risk of pneumonia during the 1-year follow-up period.

**Results:**

Incidence rates of pneumonia during the 1-year follow-up period were 4.35 (95% confidence interval (CI): 1.89∼9.64) per 100 person-years and 2.14 (95% CI: 1.31∼3.32) per 100 person-years for PC patients who did and those who did not receive treatment with GnRH agonists, respectively. The log-rank test suggested that there was a significant difference in the 1-year pneumonia-free survival rate between PC patients who did and those who did not receive treatment with GnRH agonists (*p*<0.002). After adjusting for age, monthly income, and the Charlson Comorbidities Index score, PC patients who received treatment with GnRH agonists were more likely to have been hospitalized for pneumonia during the 1-year follow-up period than PC patients who did not receive treatment with GnRH agonists (hazard ratio: 1.92, 95% CI: 1.10∼3.36).

**Conclusions:**

PC patients who received treatment with GnRH agonists had an increased risk of pneumonia.

## Introduction

Prostate cancer (PC) was the second most frequently diagnosed cancer and the sixth leading cause of cancer death in males worldwide in 2011 [1]. In Europe in 2012, it was the 3rd most diagnosed cancer after breast and colorectal cancers with 417,000 cases [2]. PC may cause pain, difficulty urinating, problems during sexual intercourse, and erectile dysfunction [3]. Androgen deprivation therapy (ADT), including an orchidectomy, estrogens, luteinizing hormone-releasing hormone (LHRH) agonists, anti-androgens, and gonadotropin-releasing hormone (GnRH) receptor agonists and antagonists, is the standard treatment for PC [4]. In particular, GnRH agonists are the most extensively used for treating PC.

GnRH agonists induce reversible hypogonadism and decrease serum levels of androgen, including testosterone and dihydrotestosterone (DHT) levels [5]. Several studies indicated that sex steroid hormones participate in communication between microorganisms and mammal hosts [6]. Effects of sex steroid hormones on diseases produced by bacteria depend on the infective species and hormone levels. In general, males of many species are more susceptible than females to infections caused by parasites, fungi, bacteria, and viruses. One major cause of sex differences in infection is differences in endocrine-immune interactions [7]. Androgen maintains a reduced expression of key elements for innate immunity and diminishes the antibacterial ability. In an animal study, Quintar et al. found a time-dependent increase in antibacterial substance β-defensins (rBD-1) and surfactant protein D (SP-D) during days 3∼7 after an orchiectomy [8]. However, GnRH agonists need to be used over the long-term for PC treatment. It still remains unclear whether or not the long-term use of GnRH agonists increases the subsequent risk of infection. According to our knowledge, no prior study has attempted to evaluate the effect of the use of GnRH agonists on the risk of infection. Therefore, in order to fill this gap in our knowledge, we examined the relationship between the use of GnRH agonists and subsequent risk of pneumonia in patients with PC using a population-based dataset from Taiwan and a retrospective cohort design.

## Methods

### Database

This retrospective cohort study used data sourced from the Longitudinal Health Insurance Database (LHID2000). The LHID2000, compiled by the Taiwan National Health Research Institute (NHRI), includes medical claims data and registry files for 1,000,000 individuals randomly sampled from all enrollees in the Taiwan National Health Insurance (NHI) program in 2000 (*n* = 23.72 million). The LHID2000, which was open to the researchers in Taiwan, was available from the NHRI (http://nhird.nhri.org.tw/date_01.html). This study is based on de-identified secondary data from the LHID2000 released by the NHRI without restrictions for research purposes. Hundreds of researchers have used this dataset to perform and publish studies [9]. The LHID2000 provides an excellent opportunity for researchers to trace the use of all medical services for these 1,000,000 beneficiaries since initiation of the NHI program in 1995. This study was exempt from full review by the Institutional Review Board since the LHID2000 consists of de-identified secondary data released to the public for research purposes.

### Study Sample

We first selected all patients who had ever received a first-time principal diagnosis of PC (ICD-9-CM code 185, malignant neoplasm of prostate) in an ambulatory care visit (including outpatient departments of hospitals and clinics) or during hospitalization from 1 January 2001 to 31 December 2010 (*n* = 2136). We excluded patients aged under 40 years, because of the very low prevalence of PC in this age group (*n* = 63). We further excluded patients who received an orchiectomy during the 1-year follow-up period (*n* = 9). As a result, 2064 PC patients were included in this study. Of the sampled PC patients, 1207 (58.5%) received treatment with GnRH agonists for more than 1 month after the index date. For PC patients who did not receive GnRH agonists, we assigned the date of receiving their first-time PC diagnosis as the index date. However, for PC patients who received treatment with GnRH agonists, we defined the first ambulatory care visit for treatment with GnRH agonists as the index date.

In this study, we individually traced each PC patient for a 1-year period to identify those who were hospitalized with a principal admission diagnosis of pneumonia (ICD-9-CM codes 480∼483.8, 485∼486, and 487.0), starting from their index date. This study also censored cases if individuals died from non-pneumonia causes during the 1-year follow-up period (124 patients died, including 96 who received treatment with GnRH agonists and 28 who did not).

### Statistical Analysis

We used the SAS statistical package (SAS Institute, Cary, NC) to perform all statistical analyses. We performed Pearson χ^2^ and *t*-tests to compare differences between PC patients who did and those who did not receive treatment with GnRH agonists, in terms of sociodemographic characteristics (age, monthly income, and the urbanization level and geographic location of the patient's residence, i.e., northern, central, eastern, and southern Taiwan). These conditions were only counted if they occurred either during hospitalization or in two or more ambulatory care claims coded 6 months before and after the index ambulatory care visit.

We also computed the 1-year pneumonia-free survival rate using the Kaplan-Meier method, and used the log-rank test to investigate the risk of pneumonia for patients who did and those who did not receive treatment with GnRH agonists. We further performed a Cox proportional hazard regression, censoring cases if individuals died from non-pneumonia causes during the 1-year follow-up period and adjusting for sociodemographic characteristics and the Charlson Comorbidities Index (CCI) score, in order to explore the association between the use of GnRH agonists and the risk of pneumonia during the 1-year follow-up period. We used a two-sided *p* value of 0.05 to determine significance in this study.

## Results

The distributions of demographic characteristics and CCI score for the sampled patients are presented in Table 1. There was a statistically significant difference in the mean age (74.2 vs. 71.9 years, *p*<0.001) and monthly income (*p*<0.001) between PC patients who did and those who did not receive treatment with GnRH agonists. In addition, there was a statistically significant difference in the distribution of CCI scores between these two groups. Patients who received treatment with GnRH agonists were more likely to have a CCI score of ≥4 than patients who did not receive treatment with GnRH agonists (*p*<0.001). However, there were no significant differences in the urbanization level, geographic location, diabetes, hypertension, and coronary heart disease between PC patients who did and those who did not receive treatment with GnRH agonists.

**Table 1:** Demographic characteristics and comorbid medical conditions of patients with prostate cancer in Taiwan, stratified by whether or not they received hormone therapy (n = 2064).

Variable	Patients receiving hormone therapy *n* = 1207		Patients not receiving hormone therapy*n* = 857		*p* value
	Total no.	Column %	Total no.	Column %	
Age (years), mean (standard deviation)	74.2 (8.5)		71.9 (11.6)		<0.001
Charlson Comorbidity Index score					<0.001
0	307	25.4	374	43.6	
1	140	11.6	102	11.9	
2	50	4.1	38	4.4	
3	258	21.4	188	21.9	
≥4	452	37.5	155	18.1	
Urbanization level					0.591
1 (most urbanized)	364	30.2	278	32.4	
2	317	26.2	230	26.8	
3	157	13.0	115	13.4	
4	202	16.7	129	15.1	
5 (least urbanized)	167	13.8	105	12.3	
Geographic region					0.097
Northern	599	49.6	439	51.2	
Central	262	21.7	211	24.6	
Southern	310	25.7	189	22.1	
Eastern	36	3.0	18	2.1	
Monthly income					<0.001
NT$1∼15,840	772	64.0	484	56.5	
NT$15,841∼25,000	352	29.1	231	27.0	
≥NT$25,001	83	6.9	142	16.5	
Hypertension	402	33.3	313	36.5	0.130
Diabetes	209	17.3	128	12.9	0.150
Coronary heart disease	178	14.8	135	15.8	0.530

Table 2 shows the incidence of pneumonia during the 1-year follow-up period among patients who did and those who did not receive treatment with GnRH agonists. In the total sample of 2064 PC patients, the incidence rate of pneumonia during the 1-year follow-up period was 3.65 (95% confidence interval (CI): 2.88∼4.56) per 100 person-years. Incidence rates of pneumonia during the 1-year follow-up period were 4.35 (95% CI: 1.89∼9.64) per 100 person-years and 2.14 (95% CI: 1.31∼3.32) per 100 person-years for PC patients who did and those who did not receive treatment with GnRH agonists, respectively. Furthermore, the log-rank test suggested that there was a significant difference in the 1-year pneumonia-free survival rate between PC patients who did and those who did not receive treatment with GnRH agonists (*p*<0.002).

**Table 2:** Crude and adjusted hazard ratios (HRs) for pneumonia among patients with prostate cancer during a 1-year follow-up period (n = 2064).

Presence of pneumonia	Total sample		Patients receiving hormone therapy		Patients not receiving hormone therapy		
	No.	%	No.	%	No.	%	
Five-year follow-up period							
Yes	73	3.5	55	4.6	18	2.1	
Incidence rate per 100 person-years (95% CI)	3.65 (2.88∼4.56)		4.35 (1.89∼9.64)		2.14 (1.31∼3.32)		
Crude ^a^ HR (95% CI)			2.23** (1.30∼3.82)		1.00		
Adjusted ^b^ HR (95% CI)			1.92* (1.10∼3.36)		1.00		

*Notes:* ^a^ Using the Cox proportional regression with cases censored if individuals died from non-pneumonia causes during the 1-year follow-up period; ^b^ Adjustments were made for patients' age, geographical location, monthly income, urbanization level, and Charlson Comorbidity Index score; CI, confidence interval.

Table 2 also displays the crude and adjusted hazard ratios (HRs) for pneumonia between PC patients who did and those who did not receive treatment with GnRH agonists. The Cox proportional hazard regression showed that the HR of pneumonia for PC patients who received treatment with GnRH agonists during the 1-year follow-up period was 2.23 (95% CI: 1.30∼3.82) compared to PC patients who did not receive treatment with GnRH agonists after censoring cases who died from non-pneumonia causes during the 1-year follow-up period. Furthermore, after censoring cases who died from non-pneumonia causes and adjusting for age, monthly income, and CCI score, PC patients who received treatment with GnRH agonists were more likely to have been hospitalized for the treatment of pneumonia during the 1-year follow-up period than PC patients who did not receive treatment with GnRH agonists (HR: 1.92, 95% CI: 1.10∼3.36).

## Discussion

To our knowledge this is the first study to report that there is a significant relationship between the use of GnRH agonists and an increased risk of pneumonia in patients with PC. The Cox proportional hazard regression showed that the adjusted HR of pneumonia during the 1-year follow-up period for PC patients who received treatment with GnRH agonists was 2.23 (95% CI: 1.30∼3.82) compared to PC patients who did not receive treatment with GnRH agonists.

We propose four possible explanations for the association detected in this study. First, GnRH agonists induce reversible hypogonadism and decrease serum levels of androgen. An androgen deficiency may induce morphological and biochemical changes in the lungs. In studies by Ojeda et al. [10], [11] they found significant morphological changes in lung parenchyma in surgically castrated rats compared to controls, similar to that observed in human lung emphysema. Using light microscopy, evident hypertrophic interstitial tissue was due to increased numbers of connective fibers and macrophagic invasion. Androgen suppression by surgical or pharmacological methods provokes significant alternations in the chemical composition of cell membranes and lung surfactants. Second, androgen induced alterations in antibiotic susceptibility and microbial growth in Plotkin et al. 's study [12]. Exposure to both testosterone and DHT resulted in an eightfold increase in *P. aeruginosa* susceptibility to tobramycin and also significantly slowed its growth. Third, in an androgen-deficient condition, compositions of gastrointestinal microflora may change to major representatives of pathogenic flora. Kosyreva demonstrated altered qualitative and quantitative compositions of the colonic luminal microflora on day 52 after surgical castration in Wistar rats, which were accompanied by an increase in the number of opportunistic bacteria [13]. Finally, androgen might increase neutrophil production. Chuang et al. collected blood cell counts from 33 advanced prostate cancer patients before and after surgical castration therapy [14]. Results showed that these PC patients' neutrophil differential counts moderately decreased after surgical castration therapy. In addition, they also proved that exogenous androgen can restore neutrophil counts in castrated mice. For the above reasons, we predict that PC patients receiving GnRH agonists will have an increased risk of infection due to an androgen-deficient condition.

This study has several noteworthy strengths. First, the large database is likely to be representative of the national population and offered a good opportunity to explore the association between GnRH agonists and pneumonia. Second, we took potential risk factors for pneumonia into consideration in the regression models. These included age, monthly income, the urbanization level and geographic location of the patient's residence, and the CCI. Finally, we evaluated the causes of hospitalization of PC patients. From censoring cases who died from non-pneumonia causes and adjusting for age, monthly income, and the CCI score, we found that PC patients who received treatment with GnRH agonists were more likely to be hospitalized for treatment of pneumonia during the 1-year follow-up period than PC patients who did not receive treatment with GnRH agonists. It consistently indicates that PC patients receiving GnRH agonists may have an increased risk of pneumonia.

This study does have several limitations that merit emphasis. First, because we identified patients with pneumonia receiving GnRH agonists after the index date from the LHID2000, the data did not enable us to know the pathogenic bacteria in the patient's blood or differences in the severity of pneumonia. Therefore, we could not evaluate what pathogen induced the pneumonia. Second, the LHID2000 data provide no information on patients' body mass index, smoking habits, alcohol consumption, physical activity, stage of disease, timing of GnRH, or nonprescription medications use. Therefore, we could not evaluate the impacts of these factors on pneumonia. Third, our results show that patients who received treatment with GnRH agonists were more likely to have a CCI score of ≥4 than patients who did not receive treatment with GnRH agonists. Since pneumonia is common among lower patients with high comorbid-condition, the causality of ADT and pneumonia cannot be easily established in the present study.

From this study, our findings indicate that PC patients receiving treatment with GnRH agonists may have an increased risk for pneumonia after adjusting for comorbid medical disorders. Although the mechanism of the present finding is still unclear, physicians should be aware of this association when they prescribe GnRH agonists in clinical settings. Further study is advised to confirm our findings and explore the underlying pathomechanism.
